# Can psychedelics enhance group psychotherapy? A discussion on the therapeutic factors

**DOI:** 10.1177/02698811231155117

**Published:** 2023-02-28

**Authors:** Polina Ponomarenko, Federico Seragnoli, Abigail Calder, Peter Oehen, Gregor Hasler

**Affiliations:** 1 Faculty of Psychology, University of Bern, Bern, Switzerland; 2 Addictology Department, Geneva University Hospital, Geneva, Switzerland; 3 Department of Medicine, University of Freiburg, Villars-sur-Glâne, Switzerland; 4 Private Practice for Psychiatry and Psychotherapy, Biberist, Switzerland

**Keywords:** Psychedelic research, group therapy, psychotherapy, therapeutic factors, psychedelic-assisted group psychotherapy

## Abstract

**Background:**

Despite the growth of psychedelic research, psychedelic-assisted group psychotherapy (PAGP) has received little attention in comparison to individual psychedelic-assisted psychotherapy models.

**Methods:**

In this article, we aim to discuss the therapeutic potential of PAGP, as well as outline existing models and the challenges of this approach. Using Irvin Yalom’s 11 therapeutic factors of group therapy as a basic framework, we analyse current literature from clinical studies and neurobiological research relative to the topic of PAGP.

**Results:**

We argue that combining psychedelic substances and group psychotherapy may prove beneficial for increasing group connectedness and interpersonal learning, potentially enhancing prosocial behaviour with direct opportunities to practice newly acquired knowledge about previously maladaptive behavioural patterns. Challenges regarding this approach include a more rigid therapy structure and potential loss of openness from patients, which may be ameliorated by adequate therapeutic training.

**Conclusion:**

We hope for this article to support clinical research on PAGP by presenting a therapeutic framework and outlining its mechanisms and challenges.

## Introduction

Social relationships are necessary for mental and physical health (Umberson and Montez, 2010). People diagnosed with psychological disorders often react more aversively to social rejection (Kumar et al., 2017) and may also encounter the stigma around mental illness in society (Feldman and Crandall, 2007). If such social dynamics of mental disorders are not actively addressed within the course of psychotherapy, this may negatively influence the development, progression and treatment of the condition of patients. Individual psychedelic-assisted psychotherapy (IPAP) has been introduced for the treatment of mental health problems (Luoma et al., 2020). However, this therapy relies on the patient’s own perspective and subjective reports of social relationship difficulties that can be replayed and better understood in the context of the therapeutic relationship, in which the therapist can help identifying the patients’ contribution to these difficulties and suggest alternative strategies. In contrast, psychedelic-assisted group psychotherapy (PAGP) allows the patient, other group members and the therapists to simultaneously experience and observe these difficulties in a safe and supportive environment. This engages multiple perspectives that may be therapeutic. In this article, we present a theoretical framework for future research on PAGP to better understand its potential benefits in treating social problems in addition to the benefits of IPAP.

In the 21st century, only three empirical studies on group therapy with psychedelics have been conducted (Anderson et al., 2020; Oehen and Gasser, 2022; Schmid et al., 2021). However, before the current wave of research, PAGP was extensively studied under the paradigm of psycholytic therapy (Passie, 1997). A recent meta-analysis identified a total of 76 cases of PAGP, all conducted between 1956 and 1995, in which a total of 700 patients in groups of up to 39 were treated with PAGP for alcoholism, psychopathy, bipolar disorder, post-traumatic stress disorder (PTSD), schizophrenia, depression, anxiety, phobias, eating disorders and addiction (Trope et al., 2019). Significant psychological and behavioural improvements were reported in most cases, especially in combination with evidence-based psychotherapeutic interventions and relatively high doses of psychedelics.

Clinical interest in PAGP’s efficacy and potential advantages is growing again (Beaussant et al., 2021). In this article, we examine the therapeutic factors of PAGP using current literature, and we outline its advantages and disadvantages in comparison with other methods, particularly IPAP. Our goal is to bring awareness to this topic and illustrate how the conceptual framework of Yalom’s 11 factors can further PAGP as an object of psychotherapy research.

### Group psychotherapy

Group psychotherapy is defined by Irvin Yalom (2005) as a social therapy which gives its members the chance to interact with others who can relate to their experiences. It is thought to ‘generate a positive, self-reinforcing loop: trust–self-disclosure–empathy–acceptance–trust’ (Yalom, 2005).

Today, group psychotherapy provides a solution to the problem of increasing psychotherapy demand by treating multiple individuals simultaneously, thereby reducing wait list delays and increasing accessibility and cost efficiency (Malhotra and Baker, 2022). While the group psychotherapy method is in itself very diverse in relation to diagnoses and ways of conduct, it is found to be particularly useful for homogeneous groups of patients with similar conditions, therapeutic goals and therapy stages (Burlingame et al., 2003; Malcolm et al., 2016; Wolgensinger, 2015). Research has demonstrated that group psychotherapy is an effective method for treating a variety of psychiatric and behavioural disorders, and experimental studies comparing individual therapy and group therapy indicate that group therapy is about as effective as individual therapy, although the great diversity of approaches leads to heterogeneous results (Burlingame et al., 2016; Cuijpers et al., 2008; Friedman, 2013).

### Psychedelic substances

‘Psychedelic’ (or ‘*mind-revealing*’) substances are a class of psychoactive drugs which produce temporary and often dramatic changes in perception, thought and emotion (Nichols, 2016). Psychedelics are ingested at various doses, ranging from sub-perceptual ‘microdoses’ to doses which produce strong altered states of consciousness. Microdoses have not yet consistently shown significant effects on well-being or in therapy (Polito and Liknaitzky, 2022; Seragnoli et al., 2021), but higher doses have been associated with lasting positive changes in psychopathology and well-being (Gasser et al., 2015; Noorani et al., 2018). Classic psychedelics, such as lysergic acid diethylamide (LSD) and psilocin, share agonist action at the 5-hydroxytryptamine 2A (5-HT2A) receptor (De Gregorio et al., 2021). Two other groups of non-classical psychedelics include dissociative anaesthetics such as ketamine and entactogens such as 3,4-methylenodioxymetamphetamine (MDMA), the latter of which produces psychedelic-like effects, predominantly an affective state of enhanced mood, profound well-being, happiness, and increased extroversion and sociability (Vollenweider, 2001). Due to its pro-social effects, MDMA may be particularly suitable for group therapy. MDMA increases synaptic levels of the monoamine neurotransmitters serotonin, dopamine and norepinephrine and blocks their reuptake, which is thought to be the primary source of its specific psychological effect (Montgomery and Roberts, 2022).

Psychedelics are considered safe for clinical use. Classic psychedelics have a very favourable safety profile, showing low physiological toxicity and potential for dependency (Gable, 2004; Hasler et al., 2004; Johnson et al., 2008; Nutt et al., 2010). Both classic psychedelics and MDMA can dose dependently increase heart rate, blood pressure and body temperature (Holze et al., 2022a; Vizeli and Liechti, 2017). They can also cause acute psychological distress, which is usually manageable with interpersonal support (Johnson et al., 2008). Serious adverse events are rare in both healthy and clinical populations (Breeksema et al., 2022), suggesting that well-trained therapists would likely be able to manage adverse effects from psychedelics in a group setting.

### Set and setting

Psychedelics interact both with the mindset of the patients, otherwise known as *set*, and the external conditions, otherwise known as *setting* (Reiff et al., 2020). Like IPAP, PAGP requires a preparatory phase to optimize these factors before administering any psychedelics. *Set* refers to beliefs, attitudes, choices and motivations, and therapists must help their patients adopt a suitable mindset before working with psychedelics (Schenberg, 2018; Winkelman, 2021). In our experience, working on the *set* in a group setting means not only that each individual establishes an intention towards introspection, but also a strong focus on the group’s intentions and processes. Conjointly, the *setting* should be organized during the preparatory phase of therapy and refers to the group’s atmosphere and environment, as well as the definition of the therapist role (Schenberg, 2018; Winkelman, 2021). *Setting* also includes the social context and any instructions given by the therapist, as well as the physical environment and the choice of music. Music is nearly ubiquitous in psychedelic therapy, even if the field still lack studies on it as a specific factor, and it can provide emotional guidance and synchronicity within the group (Winkelman, 2021). A safe and inviting atmosphere has also been shown to reduce acute psychological distress (Johnson et al., 2008) and may also help participants feel safe within the often intense and chaotic nature of sensory and emotional experiences in psychedelic sessions (Carhart-Harris et al., 2018). In addition, the presence of other group participants can increase physiological responses to psychedelics (i.e. heart rate), as well as make the subjective drug effects more intense (Bershad et al., 2016; Kirkpatrick et al., 2015).

One essential difference between conventional group therapy and PAGP may be that PAGP accelerates the therapeutic process of individuals within the group dynamic, without reducing the amount of emotional experiences (Blewett, 1971). It does so by adding emotionally intense, autobiographical and insightful psychedelic experiences into the course of therapy, which are experienced and discussed together. Thus, PAGP holds the potential to not only provide a socially supportive framework for therapy, but also to shorten recovery time, potentially reducing both human suffering and polypharmacy and drug dependency problems (Brown et al., 2005).

### Individual versus group psychedelic therapy

**Figure 1.**
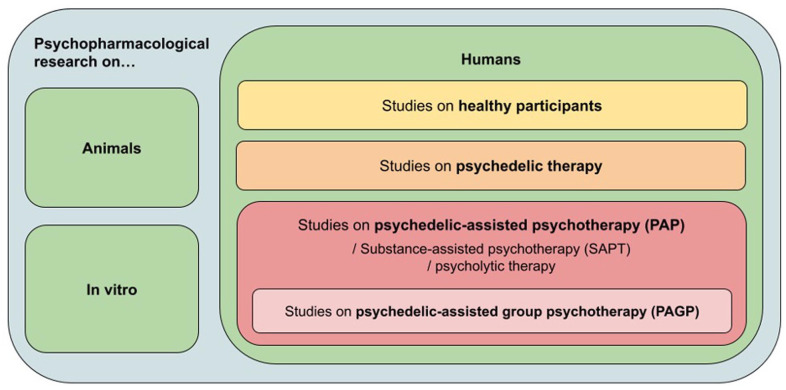
Psychedelic use in applied research.

PAGP falls into the category of applied psychedelic research, and it differs from current individual approaches (see Figure 1). Psychedelic therapy, as it is currently used in clinical trials, involves one to several high-dose sessions which are meant to produce intense and profound psychedelic experiences (Schenberg, 2018). These may contain personal insights, belief changes, emotional releases and transcendental ‘mystical’ experiences which are viewed as therapeutically beneficial (van Elk and Yaden, 2022). Psychotherapeutic integration sessions are sometimes kept to the minimum required for ensuring safety, though some researchers and clinicians have observed that psychedelic substance intake without active therapeutic accompaniment is insufficient for clinical improvement (Read and Papaspyrou, 2021). Patients often feel that their experiences with psychedelic substances are difficult to describe to those without first-hand knowledge, and if they have no one to relate to, they may withdraw socially. Such reactions are not uncommon even in healthy trial participants, and they have led to the emergence of non-professional psychedelic integration groups for past research participants (Noorani, 2019). Clearly, there is a need for more professionally accompanied interpersonal support, and group psychotherapy would be uniquely able to provide it.

IPAP sometimes addresses this problem by combining individual psychotherapy with a few introspection-focused sessions with medium- to high-dose psychedelics (Richards, 2016), often several months apart. Such interventions have been found to be successful treatments for various psychiatric disorders (Luoma et al., 2020), including treatment-resistant depression (Romeo et al., 2020), anxiety disorders (Dos Santos et al., 2021; Holze et al., 2022b), illness-related psychological distress (Gasser et al., 2015; Griffiths et al., 2016), substance use disorders (Berlowitz et al., 2019; Bogenschutz et al., 2022; Johnson et al., 2014) and PTSD (Jerome et al., 2020; Mitchell et al., 2021). IPAP entails preparatory and integrative sessions and is especially useful when the patient needs a high level of support from the clinician, for example, when working with complex disorders or a severe fear of closeness and intimacy (Oehen, 2021). However, high-dose sessions can last for many hours and are not only straining for the therapist(s), but also very costly (Beaussant et al., 2021; Teixeira et al., 2022; Vargas et al., 2021). Therefore, accommodating the IPAP model on a large scale would seem to require reconfiguring healthcare organization (Greenway et al., 2020). Medical historians have pointed out that in the mid-20th century, the laboriousness and costs of IPAP were disproportionally large compared to other treatments (Richert and Dyck, 2020).

Another existing model is ‘psycholytic therapy’, which was extensively researched in Europe in the mid- to late 20th century, with over 300 studies published on this topic (Passie, 1997). Later, it was adopted by the Swiss Medical Society for Psycholytic Therapy (SÄPT) and is still being practiced. Psycholytic therapy includes several sessions with different psychedelics, often in groups and with a varying dose range (e.g. MDMA in the first session, followed by a more intense session with LSD or a combination of both) (Passie, 1997; Reiff et al., 2020). This approach takes advantage of the different modes of substance action in combination with evolving therapeutic processes, environments and a greater access to the unconscious while discharging psychic tension (Reiff et al., 2020). The focus of this therapeutic approach is on remembering and understanding personal life experiences as well as uncovering the inner functioning of one’s mind. Preparation and integration at different intensity levels involves more personal, psychodynamic and depth psychology concepts (Jungaberle and Verres, 2008), rather than the US-American humanistic, transcendental characteristics used in IPAP, which primarily seeks to generate overwhelming *peak* experiences (Reiff et al., 2020). While IPAP and PAGP approaches can both be therapeutic, they require different forms of behaviour from the therapist and patients. In our experience, PAGP tends to bring the therapists into a more peripheral role with shared tasks and responsibilities, because mutual support and feedback often come from other group members.

Furthermore, in our experience with PAGP, we see a reduction in therapy costs for each patient because therapists can split the costs among all participants of the group. These costs include the therapists’ work time, use of the therapeutic space, and, from the patients’ perspective, the cost of transportation. The group setting is also both more rewarding and more cost-efficient than individual sessions for most problems treated with psychedelic therapy (Oehen, 2013).

## Different PAGP models

Several different frameworks for PAGP have been attempted in the past, and we would briefly outline them here. PAGP is rooted in social and ritualistic psychedelic consumption, which has been central in many cultures throughout human history (Winkelman, 2010). Today, such group ceremonies are used safely for religious purposes in both healthy and clinical populations (Halpern et al., 2008). Combinations of ritualistic and therapeutic use are also emerging, the most prominent example of which is ayahuasca-assisted therapy for addiction, in which study participants take part in ayahuasca ceremonies in retreat centres (Thomas et al., 2013).

Psycholytic therapy has been studied and practiced since the middle of the 20th century, and it has reported success in reducing psychopathology (Passie, 1997). It has not only involved psychedelic sessions in combination with regular group psychotherapy, but also other methods, including art therapy (Leuner, 1981). In one early example from 1961, PAGP was practiced with 166 heterogeneous patients in groups of 7–10 (Fontana, 1961; Trope et al., 2019). Psychoanalytic group therapy was carried out on a weekly basis and psychedelic-assisted sessions with a medium dose of LSD (50–150 μg) or psilocybin (8–12 mg) were performed monthly. After the treatment, Fontana (1961) concluded that 93 out of 166 patients were ‘cured’ or had improved significantly.

Another very different framework has been described in a study of ‘Permissive Group Therapy’, in which the author examined a group of 10 female participants diagnosed with ‘hopeless cases of chronic psychopathy, hysteria, phobic anxiety or recurrent neurotic depression’ (Spencer, 1963; Trope et al., 2019). Using a group therapy program that many would find questionable today, patients were given very high LSD doses of up to 1500 μg twice a week for 16 months. The sessions took place in a ‘child guidance play therapy room’ and allowing the patients to use the toys and dummies to represent parental figures during the sessions. The high dosages were explained by the patients’ developed resistance to LSD. After the treatment, Spencer (1963) stated that three patients had ‘improved sufficiently to require no further psychiatric help’ and with another four patients the treatment ‘helped to a fairly definite degree’.

In the 21st century, three studies have been published on PAGP, which are summarized and compared in Table 1. In the Swiss limited medical use program from 2014 to 2018, a total of 18 patients (12 women, 6 men; median age: 49 years) were treated with LSD and MDMA over several years in open group settings (Schmid et al., 2021). Drug-assisted sessions were 3.5 months apart, with 3–10 psychotherapy sessions in between them. The groups varied in size between 3 and 13 participants, with 1–3 trained psychiatrists and/or psychotherapists. The setting of the LSD- and MDMA-assisted group psychotherapy sessions consisted of a quiet meditative atmosphere, and music was played for about one-third of that time (Schmid et al., 2021). Drug administration generally started in the morning around 10 am in group sessions, during which participants were administered varying substances and doses and were encouraged to experience the first hours of the substance effects in introspective silence (Gasser, 2017). The group session finished around 6 pm, after which the participants were free to take time for themselves. An integration group session would follow the day after, before letting the patients return to their homes.

**Table 1.** Summary of the PAGP studies published since 2000.

Therapy approach	Psilocybin-assisted group therapy	LSD- and MDMA-assisted group psychotherapy	LSD- and MDMA-assisted group psychotherapy
Study design	Single-arm, open-label, pilot study	Swiss compassionate use program, prospectively designed study	Swiss compassionate use program
Substance dose	Crystalline oral psilocybin; 0.3–0.36 mg/kg	MDMA: absolute oral doses of 100 and 125 mg LSD: absolute oral doses of 100, 150, 175 and 200 μg Beginning with MDMA and transitioning to LSD in later therapy stages	MDMA: absolute oral doses of 100 and 125 mg LSD: absolute oral doses of 100, 150, 175 and 200 μg Beginning with MDMA and transitioning to LSD in later therapy stages (except when it was not trauma-related)
Total duration	Intervention: 7 weeks Follow-up: 3 months, 6.5 months, 3.2 years, 4.5 years	Ongoing therapy over a period of 4 years	Ongoing therapy over a period of 5 years
Total hours of therapy	3 h of individual psychotherapy, 12–15 h of group psychotherapy, 1 × 8-h individual psilocybin administration	Ongoing regular individual psychotherapy Mean drug-assisted sessions ± 5.7 (SD ± 0.8), Time in between sessions: on average every 3.5 months after attending 3–10 non-drug psychotherapy sessions	Ongoing regular individual psychotherapy with one of the therapists, Time in between sessions: 2–4 months
Total number of participants, group size, number of therapists	18 participants in total 18 participants per group 2 therapists per group	18 participants in total 3–13 participants per group 1–3 therapists per group	50 participants in total, Max. 12 participants per group 3 therapists per group
Inclusion criteria	(1) Gay-identified, English-speaking cisgender men >50 years old and living with HIV (2) Self-report of HIV diagnosis prior to the clinical availability of protease inhibitors (3) Moderate-to-severe demoralization Assessed by a Demoralization Scale-II (DS-II)	PTSD (61 %) Major depression (22 %) Others including (1–2 participants): anxious personality disorder, narcissistic personality disorder, OCD, dissociative disorder, bulimia, autism spectrum disorder, social anxiety disorder, psychogenic aphonia, cluster headache, life-threatening disease, substance abuse disorder, suicidal tendencies 50% of participants were on medications during treatment (antidepressants, anxiolytics, neuroleptics)	Treatment resistance in the case of trauma-related disorders: PTSD/c-PTSD/dissociative disorder (46%) Depression (20 %) Anxiety disorders (20 %) Cluster headache (10 %) OCD (4 %) ASD (4 %) Other medications (antidepressants, neuroleptics) had to be discontinued 1–2 weeks before the psychedelic session Somatic medications (antihypertensive drugs, thyroid preparations or anti-Parkinson’s medication) were continued
Exclusion criteria	Adapted from current standard criteria for psilocybin administration (Johnson et al., 2008)	Patients with psychotic disorders or unable to attend group meetings	BPD, a history of psychotic episodes, BP in patients and in first-degree relatives; acute drug dependency
Main outcomes	After 4.5 years: (1) Significant reductions in demoralization, hopelessness, death anxiety (HADS, STAI, Beck scales) (2) Significant increase: faith, spiritual well-being (3) 96 % rated the experience the single or top five most spiritually significant experience(s) of their lives (4) 100% reported ‘moderate’, ‘strong’ or ‘extreme’ positive behavioural change towards the psilocybin experience	Immediately after sessions: (1) LSD induced pronounced alterations of waking consciousness with significant increases in all dimensions and all subscales of the 5D-ASC (2) LSD produced a mystical-type experience with significant increases in all MEQ scales (3) LSD produced similar effects in patients and healthy controls (4) MDMA produced significant increases in all dimensions of the 5D-ASC except for ‘auditory alterations’; overall less pronounced effects than LSD (5) MDMA produced significant increases in all MEQ scales except for ratings of ‘internal unity’ (6) MDMA produced greater effects in patients than healthy controls	End of reporting period: (1) Clinically improved: trauma-related disorders (62%), anxiety disorders (50%), depression (40%), ASD (50%), cluster headache (20%) (2) 60% of participants terminated psychotherapy after this treatment
Source	(Anderson et al., 2020; Agin-Liebes et al., 2020)	(Schmid et al., 2021)	(Oehen and Gasser, 2022)

ASD: autism spectrum disorder; BPD: borderline personality disorder; ; HADS: Hospital Anxiety and Depression Scale; LSD: lysergic acid diethylamide; MDMA: 3,4-methylenodioxymetamphetamine; MEQ: The Mystical Experience Questionnaire; OCD: obsessive-compulsive disorder; PAGP: psychedelic-assisted group psychotherapy; PTSD: post-traumatic stress disorder; ; STAI: The State-Trait Anxiety Inventory; 5D-ASC: five-dimensional Anxiety Scale for Children.

Ongoing PAGP is still conducted in Switzerland under the Swiss limited medical use program using a model that includes group preparation, drug administration and integration. Group therapy accompanies the individual psychotherapy program of each patient and uses the so-called ‘3-day-format’ on the weekends: one group psychotherapy session takes place Friday evening, followed by the psychedelic-assisted session beginning on Saturday morning and the integration session on Sunday. While the substance-assisted sessions are held in one room for the entire participant group of up to 12 people (with three therapists), another separate room is available if a patient needs a moment of privacy with one of the therapists (Gasser, 2022; Oehen and Gasser, 2022).

Another recent study on PAGP was conducted at the University of California and consisted of three group therapy cohorts, each consisting of six gay-identified male participants (age: 50–66 years) with an HIV diagnosis and moderate-to-severe demoralization (Anderson et al., 2020). The procedure began with four group therapy sessions prior to one individual psilocybin-assisted session (0.3–0.36 mg/kg doses, 8-h duration), all of which were supported by two clinical therapists in each cohort. The day after the substance intake, each participant attended an individual integration session. In the following weeks, all participants attended 4–6 additional group therapy sessions. The total length of treatment was 7 weeks, including 12–15 h of group psychotherapy (Anderson et al., 2020). Clinically meaningful reductions in demoralization were observed following the group psilocybin session, and they persisted at a 3-month follow-up.

These studies suggest that PAGP can be effective for both homogeneous and heterogeneous patient groups, and within both fixed and open-ended time frames. In addition, different treatment models showed clinical efficacy. In the study of HIV-positive men, psilocybin-assisted therapy was conducted individually with only the integration session being in groups. In the other studies, LSD and MDMA sessions took place in the group setting, while some therapy sessions were individual. The main outcomes of the two studies that measured clinical variables showed significant improvements.

Furthermore, there are two ongoing pilot trials on psilocybin-assisted group therapy to treat cancer-associated major depressive disorder (NCT 04593563, NCT 04522804). The first is an 8-week project in which a total of 30 participants receive a single group substance-assisted session combined with one-on-one psychological support (Ross et al., 2022). The second study includes 12 participants in two cohorts and consists of a total of seven group therapy sessions, including three 2-h preparatory sessions, one 8-h psilocybin session and one 2-h integration session (Ross et al., 2022).

Outside of research studies, PAGP models have been applied to ketamine in clinical practice and have included mostly homogeneous groups of patients. Patients come once a month for 4 months, or once every 3 months for a year. After a preparatory session, the first psychedelic session involves low-dose oral ketamine to ensure safety and comfort. In the second session, the dosage gradually increases, and the third and last session includes a high-dose intense dissociative experience in which participants flexibly choose when to communicate with each other. The fourth session gives space for communal integration (Lawlor, 2021).

### Contraindications

Specific contraindications for PAGP need more investigation and have thus far differed between PAGP studies. General exclusion criteria for any psychedelic treatment currently include pregnancy or breastfeeding, cardiovascular diseases, certain medications, and a history of schizophrenia or other psychotic disorders; these are further specified elsewhere (Johnson et al., 2008). PAGP-specific contraindications mentioned in the literature include borderline personality disorder (Anderson et al., 2020; Oehen and Gasser, 2022), bipolar disorder in patients and in first-degree relatives, acute drug dependency (Oehen and Gasser, 2022) and severe lack of social skills (Gasser, 2022). In the long-term practice of PAGP in Switzerland, no other specific contraindications to PAGP have been documented. Though PAGP in Switzerland still occurs on a relatively small scale, it appears to be safe when following the abovementioned guidelines.

## Analysis

In this article, we use the term ‘psychedelic experiences’ to refer to the altered states of consciousness induced by psychedelics, including their varying phenomenological descriptions from both the ‘psychedelic’ and the ‘psycholytic’ schools. This includes general patterns of the ‘unconstrained mind’ (Lifshitz et al., 2018) embedded in subjective changes in affect, time, space, embodiment and empathy (Preller and Vollenweider, 2018), as well as what some would call ‘mystical experiences’ and the insights drawn from their contents (Yaden and Griffiths, 2021). It can also encompass the gradual softening of the autobiographical self (or ‘egolysis’) (Guss, 2022) and its more extreme version of ‘ego dissolution’ at high doses (Lifshitz et al., 2018; Milliere, 2017; Preller and Vollenweider, 2018).

When discussing PAGP, we also group two possible PAGP frameworks together: (1) traditional group therapy with the addition of substance sessions and (2) individual psychotherapy with only the substance sessions in groups (Oehen, 2021). To our knowledge, specific differences in the effectiveness of these two approaches have not been studied yet. Furthermore, the PAGP process often starts with individual psychotherapy because patients may fear opening up to the other group participants, or they may worry about the unfamiliarity of psychedelic experiences (Oehen and Gasser, 2022). A flexible approach between individual and group sessions may be useful to give patients additional support, build hope and strengthen the therapeutic alliance. We also do not distinguish between specific psychotherapeutic traditions, focusing instead on general group therapeutic factors (Gukasyan and Nayak, 2021).

In this review, we examine PAGP through the lens of Yalom’s 11 therapeutic factors within ‘Theory and Practice of Group Psychotherapy’ (Yalom, 2005). This extensive model on group psychotherapy is widely accepted and influential, and it builds the basis for many clinical group therapy frameworks (Behenck et al., 2017; Chouliara et al., 2020; Reddy, 2015). The 11 factors which play a major role in group therapy are the following:

1. Instillation of hope
2. Universality
3. Imparting information
4. Altruism
5. Corrective recapitulation of the primary family group
6. Development of socializing techniques
7. Imitative behaviour
8. Interpersonal learning
9. Group cohesiveness
10. Catharsis
11. Existential factors

Taken together, these factors represent a theoretical categorization of the curative aspects of all types of group therapy and are thought to affect patients positively when in therapeutic focus (Malhotra and Baker, 2022).

To gather information for the discussion of each therapeutic factor regarding PAGP, we focused our research on clinical trials and neurobiological research, as well as supplemental information from experienced practitioners in this field. We searched the databases of Google Scholar and PubMed from October 2021 to December 2022. Additional information was taken from selected chapters of relevant books.

## Yalom’s therapeutic factors in PAGP

### Instillation of hope

The installation of hope unfolds in two directions. First, it is the patient’s belief in their own capability for overcoming their illness. Second, it is belief in the effect of the therapy itself. Both are required for the patient to stay in therapy, and they are therefore the most crucial factors (Yalom, 2005).

Sometimes, the increased affectivity and insights derived from psychedelic sessions can bolster patients’ belief that recovery is possible. Increased belief in one’s power to recover has been seen in patients’ experiences with psilocybin and MDMA in multiple studies (Breeksema et al., 2020; Noorani et al., 2018; Watts et al., 2017). Psychedelics can also enhance autobiographical memory recollection (Baggott et al., 2016), through which patients may realize the depth, complexity and nuances of his or her personal life story. This may help patients to broaden their autobiographical perspective, bolstering both hope and motivation for recovery. In qualitative interviews conducted by Passie (2012), PAGP patients have described revisiting experiences, people and events from their past, together with an emerging sense of deep primal trust in their own capabilities and in the world at large. These realizations have been particularly associated with challenging experiences, adding a sense of curiosity in painful moments, as if patients anticipate the coming relief.

In addition, PAGP patients benefit from witnessing and empathizing with the success of other group members, which may be augmented by psychedelics’ capacity to enhance emotional empathy (Dolder et al., 2016; Pokorny et al., 2017; Schmid et al., 2014). The success of others can inspire patients to envision following a similar path and realize their own self-efficacy, similar to the effects witnessed in other group therapies (Johnson and Sullivan-Marx, 2006).

### Universality

The concept of universality addresses the patient’s feeling ‘that they are unique in their wretchedness’ (Yalom, 2005). As group members begin to disclose these concerns, they create the opportunity to relate to each other. Yalom (2005) describes this process as a ‘welcome to the human race’ experience. Its ultimate stage is the realization that there is no human deed or thought that is foreign to others.

Without the feeling of universality, there is often an increased focus on one’s own mental state, greater negative self-attribution and rumination, and reduced attention to others. This can result in social isolation and difficulties in interpersonal relationships. In sharing their experiences within a structured group environment, however, patients start to understand that others share similar thoughts, feelings and problems. As the PAGP therapist Raquel Bennett observes,

In individual treatment, people often felt very alone, that they were the only person on earth dealing with whatever problem they were living with. In the group, people quickly found that there were other people who had similar issues and challenges (Lawlor, 2021).

A recent qualitative analysis of PAGP reports a reduction in this habitual self-focus, leading to a sense of belonging and interdependence in the majority of patients (Agin-Liebes et al., 2021). This suggests that treatment with psychedelics in a group setting can help to develop the feeling of universality.

Though group therapy alone can reduce feelings of uniqueness, psychedelics may amplify this effect. Psilocybin has been shown to catalyse experiences of unity, in which people feel like they are one with other people or their surroundings (Preller et al., 2016). Psychedelics are also known for their ability to temporarily eliminate the subjective feeling of having a self, sometimes referred to as ego dissolution, which people often describe as the feeling of becoming ‘one’ with other people or the environment (Nour et al., 2016). Psychedelics may cause ego dissolution by altering the functional connectivity of the default mode network (DMN), which is implicated in self-referential processing (Carhart-Harris et al., 2016; Mason et al., 2020; Palhano-Fontes et al., 2015; Tagliazucchi et al., 2016). Without the self, there can be no exaggerated focus on one’s individual mental state. This may be particularly therapeutic in patients suffering from depression, for which perceived uniqueness of one’s suffering is a prominent feature. Depression is also hypothesized to correlate with an overly active DMN and executive network (Vollenweider and Smallridge, 2022).

Group therapy alone can increase feelings of universality, and psychedelic-induced changes in beliefs about the nature of reality can do the same. Put together, we theorize that they complement each other: Interactions with other group members offer insight into the problems they have in common, and psychedelics allow patients to temporarily step outside of themselves, both of which work to reduce focus on one’s own individual suffering.

### Imparting information

During group therapy, both the therapists and other patients may share information about mental health, mental illness and general psychological dynamics, as well as advice, suggestions or direct guidance (Yalom, 2005). These processes constitute the therapeutic factor of imparting information.

Group therapy with psychedelic substances includes an additional source of information, apart from therapists and other patients: the personal insights derived from psychedelic experiences themselves. Patients often describe these in terms of learning experiences, and they can feel like they have acquired important information about themselves, their mental habits and their capabilities (Breeksema et al., 2020; Wolff et al., 2020). As participants undergo these experiences, the psychedelic therapist can support, orient and deepen the process. This is crucial because learning experiences on psychedelics are sometimes painful; psychedelics tend to intensify therapeutic processes, occasionally leading to challenging experiences for the patient (Carbonaro et al., 2016; Roseman et al., 2018b). In group therapy, all learning experiences can be processed and shared within the group, including challenging ones, and participants can be supported by both the therapists and their peers. Because PAGP encompasses both expression-oriented phases and introspection-oriented phases within the psychedelic session (Jungaberle and Verres, 2008), patients can benefit from both.

### Altruism

According to Yalom (2005), the members of a therapy group profit not only from receiving help, but also from the intrinsic motivation to give something back. Giving tends to remedy the feeling of ‘being a burden’ and it can bring back a sense of useful contribution, value and meaning in life. The opportunity for reciprocal interactions offers a unique process to patients in group therapy, which they often use and benefit from.

Psychedelics tend to enhance prosocial feelings and behaviour. In particular, MDMA has increased self-reported feelings of sociability across multiple placebo-controlled trials, with moderate to large effects on extraversion, friendliness and trust (Regan et al., 2021; Schmid et al., 2014). MDMA may also increase emotional empathy and prosocial behaviour (Hysek et al., 2014), though some environments may not be conducive to these effects (Borissova et al., 2021). LSD and psilocybin are also known to increase emotional empathy and sociality (Dolder et al., 2016; Pokorny et al., 2017; Schmid et al., 2014), and psychedelics may make it easier to see things from another’s perspective (Thal et al., 2021).

Studies also indicate that reciprocity and altruistic behaviour are more prominent when the other person is a friend, and not a stranger (Kirkpatrick et al., 2015; Kuypers et al., 2014). In PAGP, participants have the opportunity to become familiar with each other during multiple therapy sessions and often form friendships even outside of the therapy group. In addition, Preller and Vollenweider (2019) stated that after psychedelic interventions altruistic behaviour occurs predominantly in participants with low altruism at baseline, which may be of particular interest in clinical populations, as altruism and other prosocial behaviour resulting after psychedelic therapy have been documented as long-term outcomes in a clinical addiction study (Noorani et al., 2018).

With MDMA, these effects may partly depend on action at the serotonin transporter within the nucleus accumbens (Curry et al., 2019; Heifets et al., 2019; Kamilar-Britt and Bedi, 2015; Mukai et al., 2020). In addition, both MDMA and classic psychedelics increase oxytocin levels (Holze et al., 2019, 2022c; Nardou et al., 2019). Oxytocin likely facilitates many prosocial behaviours, including altruism, cooperation, caring for others and trust (Marsh et al., 2021).

From these results, we hypothesize that in PAGP, psychedelics promote altruism in patients’ interactions with each other. Patients may experience more feelings of empathy, as well as perspective shifts and other prosocial impulses, which they can directly put into practice in the group setting with people they have come to know relatively well.

### The corrective recapitulation of the primary family group

Yalom (2005) describes the resemblance of the therapy group to a family structure, often triggering implicit memories of negative experiences in the primary family of the patient. This causes discomfort at first, but once overcome, interacting with other group members in the mimicry of their primary family patterns leads to therapeutically important realizations. While this may also happen in individual therapy, the group dynamic tends to enhance this effect, revealing initial behavioural patterns and allowing them to be re-lived in a corrective manner.

An emotionally positive psychedelic group experience, in which participants are open, caring and emotionally close to each other, has the potential to be a corrective learning experience. The group can serve as a model for a loving and supportive primary family group. This is especially beneficial for victims of childhood neglect and violence who have few memories and imprints of a positive familial atmosphere. In addition, because psychedelics improve autobiographical memory (Carhart-Harris et al., 2012; Garcia-Romeu and Richards, 2018; Healy, 2021), patients may have freer access to relevant memories concerning family relationships. Throughout PAGP, patients can develop a new standard for a healthy family structure, discovering that they are able to express and receive love and kindness both during therapy and afterwards.

From experience, Oehen and Gasser (2022) observe that ‘preconscious conflicts and ineffable childhood dramas tended to be re-enacted in the group’ and that such effects, though possible within any group therapy, can be enhanced by PAGP. They go on to describe a case study:

[. . .] after being in intensive psychotherapy for more than 1 year she had her first MDMA experience in a group setting. She labelled it an ‘emotional breakthrough’ and [experienced] positive and negative emotions of previously unknown intensity [. . .]. In the group, she befriended a fellow patient and developed a sister-transference to her. The processing of this transference reaction was very important to overcome her chronic feelings of guilt toward her biological sister and to gain independence, self-worth, and authenticity. This led to her being able to interact with her fellow patient (and the others) in a good and increasingly mature way, with clear boundaries.

However, problematic group dynamics, which reinforce dysfunctional behavioural patterns, can also occur within group psychotherapy and have also been observed in the Swiss limited medical use program of PAGP. Such dynamics could sometimes last for several sessions until they were appropriately addressed and dealt with by the therapists. Nevertheless, such problems have not thus far led to an overall negative outcome or a reinforcement of dysfunctional patterns in individual participants.

From today’s perspective, this therapeutic factor can be extended towards not only the primary family group and the individuals’ unconscious inner systems of tension or COEX systems (systems of condensed experience) (Grof, 2000), but also to any other autobiographical memories of relational nature. This could be part of the general phenomenon that psychedelics disrupt psychological habits, fixed cognitions and other constraints or ‘priors’ which limit one’s thinking (Carhart-Harris and Friston, 2019; Swanson, 2018). Patients may gain an increased awareness of avoidance tendencies in difficult social situations (Gasser et al., 2015), potentially allowing them to process frightening stimuli in a relatively unconstrained mind (Frecska, 2011), yet still within the supportive framework provided by other group members and therapists. This could allow for a more open and positive conversation about traumatic memories in the integration sessions.

### Development of socializing techniques

The development of basic social skills is a crucial therapeutic factor present in group therapy, although it varies greatly depending on group goals. To promote this, therapists can use both direct techniques (e.g. role-play) and indirect, dynamic techniques (e.g. interpersonal feedback) (Yalom, 2005).

In addition to the effects on pro-social behaviour discussed above, psychedelics may also benefit the development of social behaviour via changes in mood (Preller and Vollenweider, 2019). Psychedelics tend to enhance positive mood, as well as reduce the response to negative facial expressions and increase goal-directed behaviour towards positive compared to negative cues (Kometer et al., 2012; Preller and Vollenweider, 2019; Rocha et al., 2019; Roseman et al., 2018a; Wardle and de Wit, 2014). MDMA also enhances ratings of pleasantness for affective touch (Bershad et al., 2019) and significantly increases the emotional and social content of spontaneous speech, emotional disclosure and comfort with describing emotional memories (Baggott et al., 2016; Bershad et al., 2016). Such findings are hypothesized to lead to an increased social interest (Bedi et al., 2010), more acceptance of group interactions and increased responsiveness to therapeutic interventions from the clinician. These pro-social effects outlast the acute drug effects (Noorani et al., 2018), meaning that socializing techniques may be of benefit in the integration sessions.

Psychedelic intake may also make patients less sensitive to social exclusion. After exposure to MDMA or psilocybin, participants in one study showed reduced feelings of social exclusion in the CyberBall task, as well as a significant reduction in activation of the right anterior mid-cingulate cortex, a key region for social exclusion processing (Frye et al., 2014; Preller et al., 2016). Psychedelics may therefore reduce social pain processing, which is associated with changes in self-experience. During therapy, patients may therefore take social pain less personally, which may provide an opportunity to reflect more mindfully on social behaviour. Adding to this, MDMA interventions have been reported to reduce interpersonal defensiveness (Bedi et al., 2009; Mithoefer et al., 2011; Oehen et al., 2013), which may help patients to break out of the blaming communication style and begin to open up and reconnect to others.

### Imitative behaviour

During group therapy, patients have the tendency to imitate not only the behaviour of the therapist, but also that of other group members when learning how to address problems (Yalom, 2005). This therapeutic effect generally plays a more prominent role in the beginning of therapy and lessens after a while, as patients determine the usefulness of the mimicked behaviours and decide whether to adapt them long-term or not.

Imitative behaviour in PAGP has often been described, but its effects are not yet entirely understood. Eric Osborne, the founder of a psychedelic retreat centre, states

Within the direct experience, how many times we have seen the release of someone in the group, possibly even at a distance, that has impact on others within the group and feeling OK to just let it go. [. . .] We have instances where someone’s outburst or emotional release will trigger someone else’s. (Osborne and Townsend, 2020)

Because psychedelics can increase empathy (Holze et al., 2021; Mason et al., 2019), group members may become more aware of the distress of others in the group. If they then mimic the perceived behaviour, they may find themselves seeking an explanation for it within their own functioning. This may, in turn, help them in the process of identifying their own disturbing emotions and follows a corrective adjustment in the gradually evolving environment of the PAGP setting (Blewett, 1971).

Passie and Dürst (2008) analysed interviews of patients after PAGP and summarized multiple examples of members going into the social ‘roles’ of other members during the psychedelic interventions. Through this imitative behaviour, they were said to be exploring other gender, family or work roles, thereby empathizing even with members that seemed to have completely different situations from themselves (Passie, 2012). In summary, imitative behaviour has been documented in relation to PAGP, though more research is needed to analyse this phenomenon and its usefulness in more detail.

### Interpersonal learning

Yalom (2005) explains this complex therapeutic factor by dividing it into three main concepts. First, all members must understand the importance of interpersonal relationships for health and well-being. Second, patients should have corrective emotional experiences which disconfirm the pathogenic beliefs arising from negative memories of previous social interactions. Lastly, interpersonal learning experiences will develop into a social microcosm over time, in which group members will continuously become more comfortable with expressing themselves. As this occurs, they will begin to reveal more maladaptive interpersonal behaviour, which can then be addressed accordingly.

During PAGP, psychedelics may augment corrective emotional experiences. A recent clinical study of PAGP in patients with moderate-to-severe demoralization observed a significant reduction in attachment anxiety 3 months after psychedelic intervention (Stauffer et al., 2021). Other PAGP patients also reported overcoming previous emotional boundaries, resulting in new-found feelings of connectedness with other group members and loved ones during and after the psychedelic intervention. During the treatment and integrative therapy, nearly all participants recognized destructive unconscious habits and previously ineffective coping strategies. In semi-structured interviews, they described an augmented capacity to recognize these past patterns, and this awareness made them feel acceptance, forgiveness and relief. They also acquired a more flexible, multidimensional understanding of themselves and new self-narratives, which aided them in their interactions with other group members, as well as with loved ones (Agin-Liebes et al., 2021).

Another study on PAGP also mentions the therapeutic potential of interpersonal learning, whereby processing shame (e.g. from being a victim of sexual abuse) in the group setting seemed more difficult for some participants. Validation from other members was reportedly helpful, and it both reduced the feeling of having a unique struggle and promoted learning experiences (Anderson et al., 2020). Similarly, interpersonal support can be a powerful amplifier of therapeutic results during PAGP, positively affecting attachment security (Stauffer et al., 2021) and giving instant opportunities to act on newly acquired insights, thereby deepening the learning process (Passie and Dürst, 2008). Gasser (2022) found that PAGP can improve patients’ psychedelic experiences, particularly during difficult moments in their sessions. These difficult moments may include the emergence of negative memories, strong emotions or changes in beliefs. PAGP provides a supportive community that can help individuals navigate these personal challenges and may also highlight commonalities in their unconscious conflicts and relationship dynamics. In this way, group members may experience both a network of supportive relationships and become a role model themselves (Gasser, 2022).

Furthermore, psychedelics may amplify learning processes in general through their effects on neuroplasticity. Neuroplasticity denotes the brain’s ability to modify its connections in an adaptive manner, and it underlies learning of all kinds (Sasmita et al., 2018). Classic psychedelics, MDMA and ketamine have all been shown to lastingly enhance the growth of dendrites and synapses in the neocortex, including in areas relevant for emotional regulation (Calder and Hasler, 2022; Ly et al., 2018). Though research on learning is still sparse, there is some indication that psychedelic intake benefits certain types of learning, including reversal learning and reward learning (Buchborn et al., 2014; Gimpl et al., 1979; Kanen et al., 2022; King et al., 1974; Romano et al., 2010). Group learning processes occurring in PAGP could potentially be amplified by these physiological effects.

### Group cohesiveness

Yalom (2005) defines cohesiveness as the group therapy analogue to the therapeutic relationship in individual therapy, although it is more complex because more people are involved. A strong and positive bond, as well as the ability to constructively deal with conflict within the group, is important in altering cognitive distortions and loosening maladaptive beliefs. Group cohesiveness is seen as a precondition for the other factors to function optimally.

Cohesion develops as patients increasingly participate in the group. They begin to influence each other and allow themselves to be influenced, they listen to and accept others, they self-disclose and learn to feel secure, and they protect group norms and resist disruptions. PAGP is a long-term project, because these things take time to develop. Oehen and Gasser (2022) describe how this process might look:

Over time a core group of participants who continuously attended psychedelic-assisted sessions was formed. They also began to meet and provide mutual support outside of the therapy, and new friendships developed. The more advanced they were in their individual long-term therapeutic process the more they engaged directly in supporting and confronting their peers during the sessions in a co-therapeutic sense, as well as taking care of and serving as a model for newcomers, helping to experience what secure attachment, mutual responsibility and care for each other really mean.

This holds imperative value during PAGP, as group sessions with psychedelic substances can improve trust (Schmid et al., 2014) and the ability to connect in a social context, potentially leading to long-lasting and healthy relationships (Kettner et al., 2021; Roseman et al., 2021). MDMA in particular has been found to act as a social catalyst in group settings, amplifying facets of group connectedness (Regan et al., 2021). Blewett (1971) points out that during PAGP, interpersonal connection seems to be relatively free of distortions and empathic bonding occurs with more direct, clear communication. A frequent cause of criticism and conflict within the PAGP group is when participants start communicating inauthentically and hiding feelings during the group exchange, in which case it is often the more experienced members that begin the confrontation spontaneously to re-establish cohesiveness. It is advisable for the conflict to be declared before the psychedelic session so that the resolution can come during the sharing phase, when participants are usually more open and compassionate with each other (Oehen and Gasser, 2022).

The PAGP study by Agin-Liebes et al. (2021) also concludes that after the psychedelic intervention, group members express more feelings of openness and relaxation while being in the group, even if this had not been so just before the session. Other effects included enhanced emotional safety and more willingness to process painful emotions in the group. In addition, patients may feel closer to each other simply by virtue of having shared a psychedelic session: people often find psychedelic effects hard to describe to others who have not experienced them, and the other PAGP group members therefore have a relatively unique capacity to understand each other’s experiences (Nielson et al., 2018; Pedersen et al., 2021).

Connectedness to other members seems to come quickly for some participants, and peer support is described to be especially helpful for tolerating and understanding the content and insights derived from psychedelic experiences (Anderson et al., 2020). Gasser (2022) also elaborates on the fact that when PAGP patients start meeting outside of group sessions, it is welcomed as part of the integration process, as meeting members in ordinary life may help normalize the extraordinary experiences of the psychedelic intervention. Some patients were also relieved to realize that during the vulnerable psychedelic sessions, boundaries were still being respected and thus personal freedom was ensured, which aided the process of opening up to the group on one’s own terms (Passie and Dürst, 2008).

Summarizing the group cohesiveness effects, among others discussed above, is a PAGP patients’ testimony:

I was a very lonely and disconnected person before I joined the group. The psychedelic group experiences helped me to open up, to be close to others, to express myself, to become authentic and to build relationships. I realized that other people also had severe problems and to listen to them and watch them change from one session to the next was fascinating, enlightening and encouraging. To talk to the group about all the bad and painful things that had happened to me as a child was an extremely helpful and healing experience. I appreciated the group rules and the clear structure of the workshops. The preparation evening before and the sharing and integration the morning after the experience were very important parts of the treatment. (Oehen and Gasser, 2022)

### Catharsis

Catharsis means intense emotional discharge, and whether negative and positive, it plays an important role in the group therapeutic process. According to Yalom (2005), catharsis alone does not relate to positive outcomes; group psychotherapy following a cathartic experience is essential. It is also intricately related to cohesiveness: ‘members who express strong feelings toward one another and work honestly with these feelings will develop close mutual bonds’ (Yalom, 2005).

As psychedelic intake intensifies affectivity and embodiment (Preller and Vollenweider, 2018), it may lead to cathartic expression in clinical settings (Gasser et al., 2015). During IPAP, emotional release is associated with peak experiences and ego dissolution (Thal et al., 2021) and studies demonstrate these to be a key mediator of successive psychological changes (Bogenschutz et al., 2018; Roseman et al., 2019). Herein lies a strange paradox: though the emotions which arise under the influence of psychedelics may seem stronger than ever before, psychedelic intake also allows patients to withstand previously unbearable emotions. This has been called the ‘helioscope effect’ (Hasler, 2022): Just as a helioscope allows people to directly observe the sun, psychedelics allow patients to access and process deep traumatic memories and overwhelming emotions which were previously too painful to bear.

Recent comparative research has analysed differences between acute drug effects between PAGP and IPAP settings using data from the Swiss limited medical use program (2014–2018). Results show no significant differences (Schmid et al., 2021) indicating that cathartic effects are comparable between the two approaches to psychedelic therapy in Switzerland. To give space to and encourage cathartic processes, Gasser (2022) advises that during PAGP, the first 5 h of the acute substance effect should include little to no interaction with other group members. As the acute drug effects fade, social interactions are encouraged so that patients can process their cathartic experiences together.

There is a possibility that the expectation of great catharsis during psychedelic sessions will lead patients to fear losing control or reliving traumatic memories in front of others. We therefore advise offering the patients a sense of autonomy and control in this process and letting each person discover their boundaries within their personal experience in the psychedelic session. This can be achieved using lower to medium–high doses of psychedelics, which lowers the risk of being overwhelmed. PAGP therapists should also refrain from pushing or forcing patients into processes for which the patient is not ready.

Research shows that as the therapy progresses, members gain more control and can guide themselves more directly towards their questions and problems, as instructed by the therapist (Passie and Dürst, 2008). In our experience, it is ideal for the therapeutic process that participants have enough control to voluntarily surrender themselves to the experience, as unwillingly losing control can lead to the so-called ‘bad trips’. Maintaining some level of control seems to be as important in PAGP as the profound cathartic effect.

### Existential factors

In the process of group psychotherapy, Yalom (2005) emphasizes that members learn about the following five existential factors:

1. Recognizing that life is at times unfair and unjust.
2. Recognizing that ultimately there is no escape from life’s pain or from death.
3. Recognizing that no matter how close one gets to other people, one must still face life alone.
4. Facing the basic issues of one’s life, thus living life more honestly and being less caught up in trivialities.
5. Learning to take ultimate responsibility for the way one lives life no matter how much guidance and support one gets from others.

Many current healthcare providers see great potential in psychedelic therapy to treat existential distress (Niles et al., 2021). Clinical research suggests that IPAP can help patients with life-threatening diseases by reducing anxiety, depression, hopelessness and demoralization rapidly, substantially and lastingly (Agin-Liebes et al., 2020; Ross et al., 2016; Schimmel et al., 2022). It may also enhance the sense of meaning in life (Hartogsohn, 2018; Preller et al., 2017).

In PAGP specifically, existential factors were often expressed in a qualitative analysis of demoralized AIDS patients. Psilocybin helped the group members go from ‘trauma oriented’ to ‘growth oriented’ and improve emotional processing and regulation, leading to self-compassion and an empowering internal sense of security (Agin-Liebes et al., 2021). Ayahuasca-assisted therapy in a group retreat helped participants with substance use disorders to not only reduce their use of cocaine and alcohol long term, but also improve their sense of hopefulness, empowerment and meaning in life (Thomas et al., 2013). Contemporary existential psychologists explain these therapeutic effects of psychedelics with their ability to reduce death anxiety by forcing confrontation with mortality, reducing self-focus, shifting metaphysical beliefs, and increasing the feeling of connectedness and the meaningfulness of life (Moreton et al., 2020).

However, existential realizations should not only be attributed to the substance itself. Scharfetter (2008) addresses this issue, expressing concern about clinicians who create unrealistic expectations of psychedelic therapy. Not all suffering should become the object of therapeutic ‘elimination’, as that would be based on the hedonistic illusion of a life free of suffering. Instead, the normality of suffering should be subject to the therapeutic process. Suffering-free *spiritual* bliss is a gift sometimes given within a psychedelic session, but it cannot be the ultimate life goal. It is the strength and courage, the development of humility and acceptance, that make the pain and grief of one’s own life bearable. Having psychedelic sessions in a group context may thus help to ground people in the realization of personal responsibility by having the continuous support of multiple discussion partners, including the therapists, rather than one saviour in the form of a substance or an idealized, overpowering psychedelic therapist who promises healing and redemption.

## Patient vignette

In the fall of 2021, following previous burn-out and depressive episodes, 46-year-old Maria (fictious name) sought treatment for major depression and generalized anxiety. She had already tried different psychotherapeutic methods and had been in almost continuous treatment for 25 years, including eye movement desensitization and reprocessing, cognitive behavioural therapy (CBT), mindfulness-based therapy, psychoanalytic psychotherapy and hypnosis. Maria had also tried different forms of pharmacotherapy in conjunction with her different psychotherapies, including drugs for depression, anxiety and insomnia. During the initial consultations, traumatic episodes emerged and revealed an intense emotional neglect in her childhood coupled with maternal physical violence and verbal abuse between 12 and 15 years of age. Maria was also bullied in school, and she felt severely manipulated in her first relationship. She developed a chronic eating disorder in her youth and was having difficulties holding a stable intimate relationship, resulting in two divorces. She had three children from three different partners. Given the panel of her symptoms, she was diagnosed with Recurrent Depression (F33.2) and Generalized Anxiety (F41.1). Some symptoms related to a potential comorbidity with bulimia and C-PTSD were also noticed but not diagnosed. She started weekly ACT (acceptance and commitment therapy) talk therapy and later, after 2 months of therapy, she was recommended for a psychedelic-assisted psychotherapy group, already active and running in the therapist’s practice. Maria was psychedelic naive, did not recreationally consume any substances and was initially sceptical of joining a group setting. The most important uncertainty revolved around her fear of having to deal with the impact of other participants’ negative emotions. After the nature of the group setting and its benefits were explained, she decided to join group therapy. Maria participated in a total of four MDMA group sessions, which included between 8 and 10 participants together with three to four therapists, always in the presence of her main therapist. Overall, Maria showed a remarkable improvement in her relationship skills and participation in the group dynamic during these four sessions.

During the first two group MDMA sessions, Maria showed a marked anxiety and tension about opening up during the first exchanges with the other participants, which occurred prior to the substance intake. When having to talk at the opening and at the ending of the session, she would keep her discourse superficial, and she would avoid asking or answering questions to or from other participants. She expressed a feeling of uniqueness and of being extremely different from the other participants. Until the second session, Maria was unable to give feedback during the common discussion at the end of the session because she felt emotionally overwhelmed by the group sharing. During the third and fourth sessions, she increasingly started being more open in her communication and participating in the discussion arising from participants confronting each other. She was able to speak more about her own personal story and suffering.

During the fourth session, Maria gave feedback to others multiple times. She discussed how she had seen them change during therapy, how their comments helped her reflect on her condition, how she felt that they all were looking for the same positive feeling of being accepted and cared about, and how being heard by the group was more crucial than she previously thought. She could also finally express difficult painful emotions and wept while sharing her MDMA experience with the group. In particular, Maria realized she could stand her own emotions and would not need to avoid or run away from them anymore.

This case shows some of the group factors in action, interacting with each other, from the perspective of one participant. The interplay between the patient and the group is recognizable by her increasing participation in the group dynamic. The opening and deepening of personal processes, though at different rapidity and intensity for each patient, creates connectedness within the group. Patients who share their own experiences become increasingly able to learn from each other because they develop a common language and reference system from one session to the next. They can use this mutual psychoeducation to understand themselves, the other participants and the overall therapeutic process itself. During the fourth session in particular, Maria’s perspective shows us an interplay of instillation of hope, altruism, interpersonal learning, universality, group cohesiveness and catharsis.

After 10 months, a total of 28 sessions of weekly therapy, and four MDMA group sessions (125 mg for the first session, then 150 mg for the following three sessions), Maria reports a marked improvement in various dimensions of her quality of life. At the time of writing, Maria has been free of bulimic episodes for the past 4 months. She is relieved of the chronic anxiety she woke up with every morning for years. She reports an improvement in her sleep regulation, her relationships with her children and in her capacity for dealing with stress at work without a major breakdown. Maria also reports having milder depressive symptoms. Nevertheless, she still suffers from a feeling of shame that is at the root of her persistent depressive symptoms as well as great difficulties positively engaging in intimacy with her partner and parents.

## Challenges of PAGP

### Institutional challenges

In the emerging field of clinical treatment and research into psychedelic therapy, the individual approach is currently preferred to PAGP. One can hypothesize many reasons for this, including lack of knowledge about how to conduct PAGP, as well as the long tradition of individualistic approaches to psychotherapy in general. The implementation of PAGP into the healthcare system at psychiatric hospitals is also difficult because it requires a great deal of coordination, attention and specific knowledge about PAGP that is different from other current practices.

### Structural challenges

Some difficulties that PAGP brings may be avoided in the individual setting, one of them being the more rigid procedures. In comparison to the individual approach of IPAP, in which one or two therapists accompany one patient, our experience showed that in PAGP there can be up to four therapists who may be guiding a group at a time. The group environment may thus seem impossible to predict at first. For it to not become laborious or chaotic, it is important to outline a code of conduct and specific rules to which every participant must agree. It is therefore essential to establish a balance between individual and group processes, which requires good choices about which patients to include. Time management and session schedules are less flexible, and therapists must be mindful of individual needs regarding stillness, talking and movement, as well as substance-related openness. However, given a strong group cohesiveness, experienced PAGP members tend to actively maintain boundaries and group rules in a mostly calm and open manner, which should be encouraged. Faced with this challenge, Spencer (1963) at first allowed the patients to freely do as they wish during the psychedelic sessions, stating that

Certainly the patients in this group needed to pass through a phase in which they could do exactly as they wished and still retain the therapists’ love before they could pass on to a more mature phase of accepting the demands of society upon them.

During psychedelic sessions, coordinating individual differences in dose and effect may be laborious, as well as disturbances due to noise and restlessness. In general, we can say that a higher degree of illness requires more active guidance of the patient. However, it also can be especially difficult to integrate distinctly egocentric individuals within the group. Generally, there is also the concern of patients being less open in the group setting compared to the individual setting, as they could be worried about other people’s reactions during the session. This could lead to interferences or a loss of focus within the intrapersonal process.

### Challenges with therapeutic factors

Challenges related to PAGP are similar to those found in conventional group therapy. Before starting therapy, therapists should carefully screen patients to determine eligibility. External factors, such as acute stress or moving, can lead to early dropout (Yalom, 1966). It is also important to not create false expectations that the therapy is beneficial for everyone, as some patients may prefer individual therapy or request a disproportional amount of personalized attention.

A lack of universality can sometimes lead to ‘early provocateurs’ (Yalom, 1966), who exhibit aggressive behaviour and then leave due to anxiety. Others may develop a feeling of not fitting in, which is called group deviancy and is accompanied by a lack of psychological sophistication and interpersonal sensitivity. This can damage the group cohesion process if not addressed by the therapist. Providing individual sitters for these patients can help them feel supported.

Without the corrective recapitulation of the primary family group, problems with intimacy may arise, such as schizoid withdrawal, maladaptive self-disclosure or unrealistic demands for ‘instant intimacy’ (Yalom, 1966). When socializing techniques are not developed with the guidance of the therapist, complications can also arise through subgrouping, in which patients who feel left out create their own subgroups within the therapy group, damaging the group cohesion process.

Interpersonal learning processes can be disturbed when patients are strongly affected by hearing the problems of other group members, causing fear or upset. This can have a lasting impact on the group atmosphere, and therapists should address these reactions individually and encourage an accepting mindset within the group.

Another point addressing the interpersonal exchange in PAGP is that some authors have argued that psychedelic experiences can seem ineffable (Nielson et al., 2018; Nielson and Guss, 2018; Pedersen et al., 2021). They can be vastly different not only inter- but also intra-individually, potentially creating a greater feeling of isolation or uniqueness amongst group members rather than the desired therapeutic outcome. This especially concerns difficulty describing the experience in integration sessions, but also portends difficulties with properly informing naïve patients about what they can expect in a psychedelic session (Johnson et al., 2008). It therefore generates considerations regarding the correct preparatory approach of *set* and the establishment of a shared vocabulary. More research on PAGP is needed to identify the most beneficial therapeutic context to introduce the psychedelic group experience to the patient. Focusing more closely on the influence of an interpersonal setting on psychedelic intake may shed light on further therapeutic factors.

Depending on numerous variables in patients’ predisposition, dosage, set and setting, the ‘peak psychedelic experience’, meaning the moment the effects are perceived to be the most intense, is not always pleasant (Passie and Dürst, 2008). ‘Bad’ or ‘challenging’ experiences endure critical discussion about their therapeutic efficacy, as some argue that they can lead to therapeutic benefits, and others point out that they can have the potential to be damaging or provoke undesirable changes in core beliefs (Carbonaro et al., 2016; Greif and Surkala, 2020; Roseman et al., 2018b). This may lead the patient to lose hope with the therapy approach or create disturbances within the group. An essential aspect of PAGP is that challenging experiences are well supported as they happen, as well as thoroughly discussed in a social setting after they subside. Giving psychedelics too frequently without sufficient integration of the experience into daily life may also result in delusions and disappointment in the long run (Oehen, 2013).

Finally, we echo some problems mentioned in an earlier PAGP study (Spencer, 1963). Treating all patients in the group equally is important to lessen the feeling of uniqueness and strengthen the group cohesiveness. Adding to this, not all participants will always be willing to participate in the group process, which can affect the therapeutic factors in general. Lastly, therapists need to be prepared for the potential physical acting out of a cathartic expression.

## The PAGP therapist

In PAGP, it is not only the substance that has therapeutic effects, but also the accompanying psychotherapy, for which correct training of the therapist plays a crucial part. Phelps (2017) outlines six basic competencies of the psychedelic therapist: empathetic abiding presence, trust enhancement, spiritual intelligence, knowledge of the physical and psychological effects of psychedelics, self-awareness and ethical integrity, and proficiency in basic and complementary psychotherapeutic techniques. As PAGP usually involves multiple therapists, differences in therapeutic styles should be discussed beforehand to avoid incoherent processes. Current models of successful PAGP suggest using meaning-centred therapy (Beaussant et al., 2021) or the Accept, Connect, Embody model, based on ACT (Teixeira et al., 2022), as well as the theoretical integration of attachment theory, mindfulness-informed and relational approaches (Agin-Liebes et al., 2021) and synergistic therapeutic mechanisms (Thal et al., 2021).

While conventional group psychotherapy often has a specific focus or goal (Teixeira et al., 2022), in PAGP the focus lies broadly within the experience of the psychedelic sessions, the interpretation of its personal insights, and the integration of the aforementioned psychological processes. The personal issues that may come up during this therapy may vary a lot within the group. Therefore, therapists need to be able to flexibly observe and reframe events to promote the core processes of the group, while also being able to bring the group’s attention to a particular issue a given patient is going through. This is possible during the group integration discussions, in which patients share their own subjective experience with the group.

Clinicians should also be aware of the intensity and length of psychedelic sessions. Sufficient time needs to be spent with every group member to support connectedness, comfort and therapeutic progress. This goal is complex and especially difficult in a group setting. Therefore, therapist cooperation is advised (Johnson et al., 2008), preferably involving therapists of different genders (Passie and Dürst, 2008).

Specific interventions by PAGP therapists are particularly required in situations where patients show difficulties during psychedelic sessions, such as being emotionally overwhelmed, dissociating, freezing or resisting. Verbal interventions should be kept to a minimum until the late phase of the session since they risk leading the patient away from the embodied emotional experience. Alternatives may include humming, singing, storytelling and reciting poems (Oehen, 2013). In addition, patients with deep rooted trauma sometimes benefit from emotionally corrective and restructuring interventions involving body contact and appropriate touch included in group settings (Oehen, 2013; Oehen and Gasser, 2022; Spencer, 1963), whereby we do not differentiate between different ways of touch in individual and group settings. After having clearly obtained consent for this aspect of the therapy during preparation with the patient, the PAGP therapist may touch the public parts of the patient’s body during a challenging moment of the experience. Some examples of this include contact between the hand of the therapist and the hand, head or shoulder of the patient.

Another responsibility of the psychedelic therapist is to be mindful of transference in the interactions between therapists and group members, as well as between the group members. Transference is a concept derived from psychoanalysis and refers a tendency for representational aspects of important relationships, such as feelings, desires and expectations of one person, to be consciously or unconsciously applied to others (Levy and Scala, 2012). In the group therapy setting, transference can become more complex, less controllable and potentially reinforced by unprofessional behaviour of other group participants, including unwanted intimacy and sexuality (Peluso et al., 2020). Therefore, the observance of boundaries and clear and unambiguous group rules as well as a transparent therapy concept are indispensable.

### The therapists’ self-experience with psychedelics

In the 18th and 19th centuries, clinicians were encouraged to have self-experiences with psychoactive substances before administering them to patients. In contrast to such controlled self-experiments, today’s scientific experiments that are more carefully designed often do not provide enough detailed information about the subjective experiences (Passie and Brandt, 2018). However, therapists must understand the psychoactive effects to know how to convey psychedelic therapy to their patients. If a therapist expects overly idealistic outcomes from the work with psychedelic substances, he or she may not only overstrain the patient, but also abandon the attitude of a supportive psychotherapist (Noorani and Martell, 2021). In conveying the particular nature of PAGP, we argue that the therapist can gain the most accurate knowledge of how to understand and describe the complexity of psychedelic states by experiencing psychedelics themselves. In the same way as any psychotherapist has the duty, ethically and administratively, to have undergone psychotherapy, we believe that a PAGP therapist would benefit from their own supervised psychedelic intake in a therapeutic group setting, which should be part of the obligatory psychotherapeutic training (Gasser, 2022; Oehen, 2013). As Oehen and Gasser (2022) have stated, ‘In our view, psychedelic therapists should have experienced drug-induced altered states of consciousness in a therapeutic setting themselves in order to fully appreciate and understand the mechanisms of action, possibilities, limitations, and pitfalls of the process’.

Since a great deal of the psychotherapy work revolves around promoting the patient’s self-reflective and self-regulating skills, psychedelic therapists will be better able to guide the patient in discovering and understanding how their mind works if they can refer to their own subjective experience. This is even more important when dealing with psychotherapies that used altered states of consciousness, like IPAP and PAGP. This is also the case for mindfulness-based CBT, which is based on the patient learning meditation techniques. The mindfulness therapist in training is asked to practice mindfulness meditation and learn from personal experience in order to be able to best implement this therapy and understand patients’ experiences with it (Crane and Kuyken, 2013). In addition, patients in altered states of consciousness sometimes pick up on extremely subtle cues to the therapist’s current mental state. A therapist who is not authentic and aware of his or her own weaknesses and unresolved issues will not always be able to fool patients, which may get in the way of the therapeutic process (Oehen, 2013).

However, therapist experiences with psychedelics may not be currently feasible, since psychedelics are still scheduled as dangerous, illegal drugs under the UN Conventions and many governments’ drug laws (Nutt and Carhart-Harris, 2021). Nowadays, an aspiring psychedelic psychotherapist could feel forced to do something that is illegal or at least stigmatized, even in countries with legal psychedelic drugs (Nielson and Guss, 2018). If there is no access to an organized therapeutic setting, taking psychedelic substances alone risks either overburdening the therapist or being perceived as superficial. Guided psychedelic sessions in an illegal context could also create a stigma from the patient perspective, as they may worry about their therapist engaging in illicit activities, which could negatively impact trust and the therapeutic alliance (Nielson and Guss, 2018).

There have been only a few documented cases of psychedelic sessions conducted within a therapist training program. In the 1970s, one study examined 203 mental health professionals undergoing experiences with LSD as a part of their training in the United States. However, the data were never systematically analysed or published (Nielson and Guss, 2018). Individual therapist experiences have been documented within the MDMA-assisted therapy training from the Multidisciplinary Association for Psychedelic Studies, and those therapists reportedly felt more prepared to conduct their therapy sessions (Nielson and Guss, 2018). Further research into therapist efficacy is needed to evaluate this result. Regarding PAGP, one psychedelic therapist training at the Imperial College London has successfully piloted a study on potential psychedelic therapists undergoing a voluntary group experience with psilocybin (Nutt and Carhart-Harris, 2021). These are first steps towards a wider acceptance of guided psychedelic sessions in a PAGP therapy training program, but more research into outcomes from these trainings is needed.

## Limitations and future considerations

One limitation of this article is a general lack of clinical studies on PAGP, despite the fact that this therapeutic framework is already practiced in some countries. Future research could consider comparing conventional group therapy and IPAP to PAGP to determine more similarities and differences in method and outcome. Within clinical research, a greater focus could be placed on challenges and problems during the PAGP process, as this helps to improve the method over time. In addition, the standardization of the psychotherapeutic techniques used in trials with psychedelic drugs has been insufficiently studied.

Furthermore, no direct causal relations can be made from neurobiological findings to therapeutic outcome; we only use this information to demonstrate potential connections. Future research could benefit from studying neurobiological changes following PAGP, as well as predictors of PAGP treatment response.

Future research on PAGP could also include long-term assessments of therapeutic effects using both quantitative and qualitative approaches and comparing PAGP to other forms of therapy, such as IPAP and conventional group psychotherapy. Potential selection bias should also be considered when dealing with participants with prior experience with psychedelics. Furthermore, the specifics of different PAGP settings and therapeutic approaches should be investigated with the aim of creating internationally recognized guidelines, as well as improving the cost–benefit ratio of psychotherapeutic treatment.

## Conclusion

The aim of this paper was to examine PAGP through the lens of Yalom’s 11-factor framework for group therapy. We synthesized existing literature that outlines the potential effectiveness and challenges of PAGP. We hope that this information provides a conceptual base for more clinical research on PAGP, as well as its potential implementation in treatment centres.

In summary, we conclude that psychedelics may enhance important group therapeutic factors. Psychedelic substances can broaden the autobiographical perspective, allowing patients to re-process their personal experiences. They also seem to reduce self-focus and enhance prosocial behaviour, enabling deeper empathy and connection between group members. Psychedelic-assisted therapeutic sessions also support patients in processing frightening stimuli in the new social context, and enhanced learning ability may give them the opportunity to improve their social skills directly with the support of other group members. Psychedelics can be seen as social catalysts, amplifying group connectedness. In a well-guided setting, the intense cathartic effects of these substances are maintained even within the group experience. However, PAGP also has a relatively rigid structure with clear rules. Taken together with its social context, this may lead individuals to become less open and experience difficulties during the psychedelic session. The ineffability of the experiences may also prove challenging, though this can be overcome by adequate therapeutic training. The art is thus to maintain a clear, binding structure and orderly flow while being as open as possible to the experiences of the participants.

In sum, we conclude that psychedelic substances can be powerful vectors of interpersonal psychotherapy, and PAGP has particular therapeutic potential due to its increased accessibility and suitability for treating interpersonal issues.
